# A precision image-guided model of stereotactic ablative radiotherapy for hepatocellular carcinoma

**DOI:** 10.1242/dmm.052301

**Published:** 2025-05-19

**Authors:** Stephanie May, Katrina Stevenson, Bashaer Alqarafi, Kyi Lai Yin Swe, Algernon Bloom, Agata Mackintosh, Miryam Müller, Anastasia Georgakopoulou, Thomas M. Drake, Christos Kiourtis, Saadia A. Karim, Colin Nixon, Barbara Cadden, Aileen Duffton, Derek Grose, David Y. Lewis, Karen Blyth, Anthony J. Chalmers, Thomas G. Bird

**Affiliations:** ^1^ Cancer Research UK Scotland Institute, Glasgow G61 1BD, UK; ^2^ School of Cancer Sciences, University of Glasgow, Glasgow G61 1QH, UK; ^3^ Cancer Research UK Scotland Centre, Glasgow G61 1BD, UK; ^4^ Department of Clinical Oncology, Beatson West of Scotland Cancer Centre, Glasgow G12 0YN, UK; ^5^ Centre for Inflammation Research, University of Edinburgh, Edinburgh EH16 4UU, UK; ^6^ CRUK Scotland Centre, Edinburgh EH4 2XR, UK

**Keywords:** Hepatocellular carcinoma, Stereotactic ablative radiotherapy, Preclinical model, Multimodal therapy, Cancer, CT imaging

## Abstract

Liver tumours, both primary and metastatic, are diseases of unmet clinical need. Hepatocellular carcinoma (HCC), the most common primary liver tumour, like many other cancers, can be treated by stereotactic ablative radiotherapy (SABR), reducing off-target effects of radiation on local anatomical structures. However, integrating all the necessary components for stereotactic irradiation of HCC in murine models has not yet been reported. Here, we provide the development and detailed characterisation of a murine SABR model combining magnetic resonance imaging- and computed tomography (CT)-guided delineation of the tumour, together with CT-guided liver tumour radiotherapy. The model enables accurate delivery of clinically relevant doses of radiotherapy with good tolerability and on-target tumour responses in models with otherwise universally progressive disease. The development of this preclinical modelling platform paves the way for its integration into multimodal therapeutic and mechanistic testing in preclinical murine models of metastatic and primary liver tumours, including HCC.

## Teaser

**Editor's choice:** Liver tumours are diseases of unmet need and targets for clinical radiotherapy. We describe the development of a murine model platform facilitating image-guided stereotactic irradiation of liver tumours.

## INTRODUCTION

The liver is a common site for tumour metastasis (e.g. colorectal cancer, pancreatic cancer, melanoma) and is also the site of primary tumour formation, with hepatocellular carcinoma (HCC) being the most common primary liver cancer. Both primary and metastatic liver cancer are a major global unmet need. HCC is the third most common cause of cancer-related death worldwide, and its incidence continues to rise (Rumgay et al., 2022; Burton et al., 2021). Increasing incidence is driven, particularly in higher-income countries, by lifestyle factors including obesity and excess alcohol consumption, and disproportionally affects the most socioeconomically deprived (Llovet et al., 2021; Rashid et al., 2024; Karlsen et al., 2024). Prognosis for patients with HCC is among the worst for any type of cancer, reflecting that many patients present with late-stage disease, and systemic therapies generally offer only marginal benefit (Llovet et al., 2021).

Radiotherapy is one of the most commonly used cancer therapies in the clinic, being delivered to over 50% of cancer patients (Ahmad et al., 2012; Baskar et al., 2012). Stereotactic ablative radiotherapy (SABR) has provided new opportunities for high-precision image-guided radiotherapy, which have enabled delivery of ablative doses to target volume, while minimising the dose received by normal tissues. Improved accuracy and precision – through patient preparation, immobilisation, advanced imaging and motion management – have improved SABR treatment delivery for challenging disease sites, including the liver (Mathew and Dawson, 2021).

SABR has recently been approved for advanced, but locally confined, HCC (NHS England, 2020). It offers satisfactory local disease control in the short term and is associated with fewer serious adverse events than other treatment options, such as systemic chemotherapy and conventional radiotherapy (Eriguchi et al., 2021; Shanker et al., 2021; Liu et al., 2023). Nonetheless, SABR is rarely curative, and patients often experience recurrent or progressive tumour growth. However, SABR offers a treatment for patients who may not be suitable for other therapies. The stereotactic, normal tissue-sparing nature of SABR is particularly important in HCC as patients typically have underlying liver cirrhosis and/or hepatic dysfunction, so minimising damage to the non-tumoral liver is crucial. Now that SABR is part of the therapeutic armamentarium for liver cancer, there are significant opportunities for its integration with other forms of treatment (including immunotherapy) to optimise therapeutic regimes and improve outcomes for patients. Understanding how HCC responds to SABR will inform the design and delivery of multimodal treatment and could provide opportunities for individualised treatment strategies.

Accurate and clinically relevant preclinical animal models are needed if we are to understand the effects of radiation therapy on liver cancers and the adjacent normal tissues. The mouse offers the most widely used preclinical animal model for liver cancer, and, with the increasing human relevance of genetically engineered mouse models in liver (Llovet et al., 2021; Barcena-Varela et al., 2024; Müller et al., 2025) and other cancers [e.g. colorectal (Jackstadt and Sansom, 2016)], this species offers an ideal platform to study radiation effects. As yet, however, no model systems have been reported in which radiotherapy is accurately delivered to tumours within the rodent liver. Models of stereotactic radiotherapy to other organs and tumour types include the brain (Rutherford et al., 2019), lung (Herter-Sprie et al., 2014) and pancreas (Tesson et al., 2024). Although individual elements of HCC models have progressed – specifically, (1) reliable tumorigenesis generating tumours of sufficient size and penetrance to be differentiated from the background liver (Leslie et al., 2022; Zhou et al., 2023), (2) identification of HCC with preclinical imaging modalities (Zhou et al., 2023) and (3) targeting the liver with stereotactic radiotherapy (Kabarriti et al., 2019) – to date, a lack of integration of these components has prevented the creation of an effective preclinical model platform for targeting HCC with SABR.

To address this challenge, a murine model requires a radiologically definable liver tumour, a precision stereotactic irradiation platform and a model in which outcome is dependent principally upon the tumour being targeted by radiotherapy. HCC is readily detectable and can be diagnosed confidently in patients through the use of contrast-enhanced cross-sectional imaging [e.g. magnetic resonance imaging (MRI) or X-ray-based computed tomography (CT)]. Clinically, intravenous (i.v.) contrast is used in diagnostic hepatic radiology, both with MRI and CT (Chernyak et al., 2018). The dual blood supply to the liver from both the hepatic artery and portal vein enables phase contrast discrimination between non-tumoral liver tissue and cancer, even using extracellular contrast agents. This is due to tumours being predominantly supplied by the hepatic arterial circulation, whereas the portal venous system dominates supply to the rest of the liver. Furthermore, a number of hepatocyte-specific contrast agents have been developed, facilitating liver parenchymal delineation in both CT and MRI (Zhang et al., 2022). Integrating these agents into preclinical models of HCC SABR has not been achieved to date, however.

Here, we have established a new preclinical platform through the integration of contrast-based CT detection of intrahepatic tumours together with stereotactic radiotherapy of tumours in an immunocompetent murine model of HCC. Through optimisation of an orthotopic transplantation model, and by testing a number of contrast-enhanced CT approaches, we have opened up the potential for integrating stereotactic radiotherapy in mechanistic and functional studies in liver cancer, including multimodal therapies.

## RESULTS

### Optimisation of an orthotopic liver tumour model for SABR

**Fig. 1.**
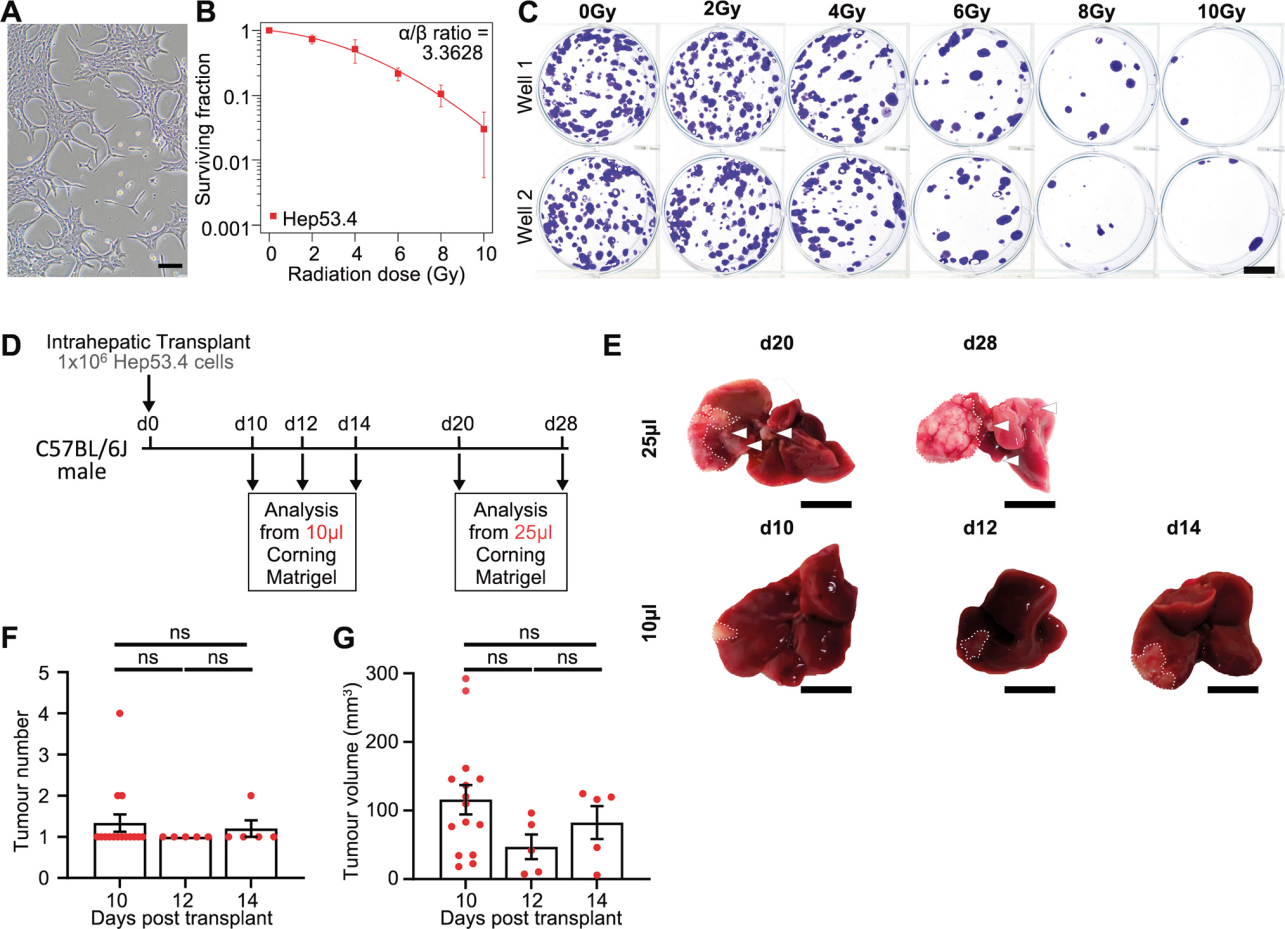
**Optimisation of a monofocal orthotopic hepatocellular carcinoma (HCC) murine model.** (A) Microscopic brightfield image of the Hep53.4 murine HCC cell line used throughout this study. Scale bar: 100 µm. (B) Clonogenic survival of Hep53.4 cells treated with X-ray radiation relative to non-irradiated (0 Gy) controls. Points represent mean surviving fraction of *N*=4 independent experiments (different passages of Hep53.4 cells) and were fitted using the linear quadratic model. α/β ratio=3.3628. Note: a fifth experimental repeat was excluded from the analysis owing to clumping together of colonies (at 0, 2 and 4 Gy), preventing accurate quantification of surviving clones. (C) Representative images of clonogenic assays of Hep53.4 cells irradiated with increasing doses of X-ray radiation as presented in B. For 0-4 Gy, 500 cells per well were seeded; for 6-10 Gy, 1000 cells per well were seeded. Scale bar: 1 cm. (D) Timeline schematic of the orthotopic HCC model optimisation based on an intrahepatic injection of Hep53.4 HCC cells into C57BL/6J mice. One million single Hep53.4 cells were implanted into a lobe of the murine liver in either 10 µl or 25 µl Matrigel and sampled at different times post-transplant to assess tumour growth. d, days post-transplant. (E) Representative images of livers from the 10 µl and 25 µl orthotopic HCC model at different timepoints post-intrahepatic injection. Dotted white lines indicate the orthotopic tumour boundary. White arrowheads indicate additional tumours growing outside the primary tumour area. Scale bars: 1 cm. (F) Quantification of tumour number at days 10, 12 and 14 post-intrahepatic injection in the 10 µl orthotopic HCC mouse model. *N*=15, *N*=5 and *N*=5 mice at days 10, 12 and 14 post-transplant, respectively. Kruskal–Wallis test with Dunn's multiple comparisons test; ns, not significant, *P>*0.05. Data shown as mean±s.e.m. (G) Quantification of total tumour burden (mm^3^) at days 10, 12 and 14 post-intrahepatic injection in the 10 µl orthotopic HCC mouse model. *N*=15, *N*=5 and *N*=5 mice at days 10, 12 and 14 post-transplant, respectively, as for F. One-way ANOVA with Holm-Šídák's multiple comparisons test; ns, not significant, *P>*0.05. Data shown as mean±s.e.m.

As the first step in developing a SABR model for HCC in the immunocompetent mouse liver, we selected the Hep53.4 HCC cell line, a classical model of murine HCC, which was originally generated from a C57BL/6 mouse that developed liver cancer following diethylnitrosomine administration (Kress et al., 1992). We and others have previously characterised this cell line, including whole-genome sequencing, transcriptomic analysis in the orthotopic setting, and documentation of therapeutic responses in both subcutaneous and orthotopic transplant models (Kress et al., 1992; Esteban-Fabró et al., 2022; Leslie et al., 2022, 2023; Zabransky et al., 2023; Wang et al., 2024). Notably, the Hep53.4 line is sensitive to immunotherapy in an orthotopic transplant model within the background of the healthy liver (Leslie et al., 2022). We first measured the radiosensitivity of Hep53.4 cells *in vitro*, using the gold-standard clonogenic survival assay. Hep53.4 cells were moderately radiosensitive, with <5% surviving fraction at 10 Gy and an α/β ratio (a measure of the relationship between clonogenic survival and radiotherapy dose per fraction used in the linear quadratic model) of 3.36 (Fig. 1A-C).

Next, we optimised an orthotopic model using this cell line. Initially, we performed orthotopic injection using 1×10^6^ cells delivered in a 25 µl Matrigel suspension, in line with previous protocols (Zabransky et al., 2023; Brown et al., 2018) (Fig. 1D). Pale tumours at the site of injection were observed 21 days after transplant (Fig. 1E). However, this model frequently presented with multi-focal tumours, including in the liver distant to the transplant injection site, suggesting intrahepatic seeding, as has been observed and discussed previously (Brown et al., 2018; Li et al., 2020; Sawada et al., 2002; Leslie et al., 2022). Because monofocal tumours are best suited for long-term studies in which focal radiotherapy is applied to the index tumour with curative intent, we refined the model by reducing the injection volume to 10 µl. This reduced the incidence of multifocal disease, with the majority of animals having single lesions at the site of injection (Fig. 1E,F). Notably tumours were universally present by 10 days, with an average volume of 115.8 mm^3^ (range, 18.38-292.3 mm^3^) (Fig. 1G), and hence the 10 day timepoint was selected for subsequent integration with SABR.

### CT imaging of orthotopic liver tumours

**Fig. 2.**
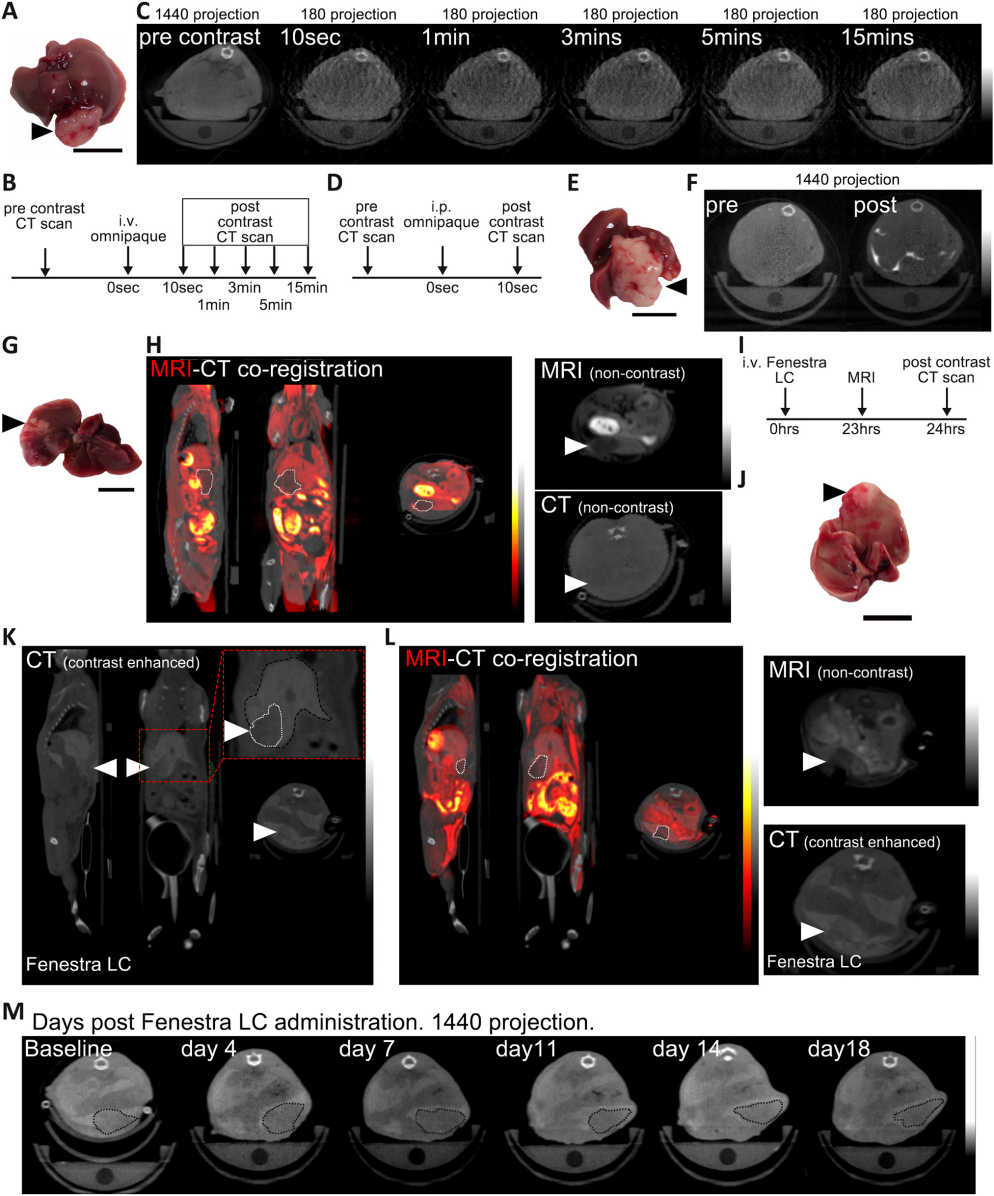
**Contrast-enhanced computed tomography (CT) image detection of orthotopic liver tumours.** (A) Macroscopic image of the liver and tumour (black arrowhead) from the 25 µl orthotopic Hep53.4 murine model used in intravenous (i.v.) administration of Omnipaque contrast agent in B and C. *N*=1 mouse. Liver/tumour were harvested 11 days after CT imaging (30 days post-transplant). Scale bar: 1 cm. (B) Timeline schematic of the non-invasive CT scans pre- and post-i.v. administration of Omnipaque contrast agent to a tumour-bearing orthotopic HCC mouse. i.p., intraperitoneal. (C) Transverse plane images of CT scans, built from 180 or 1440 projections as indicated, of a tumour-bearing HCC mouse prior to and at different times after i.v. administration of Omnipaque. CT density scale indicates the minimum and maximum range. All CT scans have been captured using the same window level. *N*=1. (D) Timeline schematic of the non-invasive CT scans pre- and post-i.p. administration of Omnipaque contrast agent to a 25 µl tumour-bearing orthotopic HCC mouse. (E) Macroscopic image of the liver and tumour (black arrowhead) from the 25 µl orthotopic Hep53.4 murine model used for (i.p.) administration of Omnipaque contrast agent in F. Liver/tumour harvested on the same day as imaging, 29 days post-transplant. Scale bar: 1 cm. (F) Representative transverse plane images of CT scans, built from 1440 projections, of a tumour-bearing HCC mouse prior to and after i.p. administration of Omnipaque. CT density scale indicates the minimum and maximum range. CT scans have been captured using the same window level. Representative of *N*=3 mice. (G) Macroscopic image of the liver and tumour (black arrowhead) from the 25 µl orthotopic Hep53.4 murine model used for co-registration of magnetic resonance imaging (MRI) and non-contrast CT scans for tumour identification in H. Liver/tumour harvested on the same day as imaging, 20 days post-transplant. Scale bar: 1 cm. (H) Representative images of co-registered T1-weighted MRI (hot colour scale, right of left panel)-CT (greyscale, right of left panel) scans of a 25 µl orthotopic tumour-bearing HCC mouse without contrast agent. White dotted lines delineate the liver tumour identified by MRI. Sagittal, coronal and transverse planes are shown from left to right in the left panel. In the right panel, transverse planes of the MRI (top)/CT (bottom) scans show clear demarcation of the tumour (white arrowhead) on MRI, which is not visible on the non-contrast CT. MRI/CT intensity scales in greyscale show the minimum (bottom) and maximum (top) range. *N*=3 mice. (I) Timeline schematic of i.v. administration of Fenestra LC contrast agent with non-invasive MRI-CT imaging in tumour-bearing orthotopic HCC mice. (J) Macroscopic image of the liver and tumour (black arrowhead) from the 10 µl orthotopic Hep53.4 murine model used for i.v. administration of Fenestra LC contrast agent in K. Liver/tumour harvested on the same day as imaging, 10 days post-transplant. Scale bar: 1 cm. (K) Representative 1440 CT images of an orthotopic tumour-bearing HCC mouse (10 µl model from J) 24 h post i.v. administration of Fenestra LC. Sagittal, coronal and transverse planes are shown from left to right, with the inset (dashed red line) showing the coronal plane with contrast enhancement in the liver (black dashed line) and delineation of the tumour (white dotted line), which is not enhanced by the contrast agent. White arrowheads indicate the tumour in all CT planes. CT density scales in greyscale show the minimum (bottom) and maximum (top) range. *N*=10 mice. (L) Representative co-registration of T1-weighted MRI and Fenestra LC contrast-enhanced CT images. Representative images of co-registered MRI (hot colour scale, right of left panel)-CT (greyscale, right of left panel) scans of a 10 µl orthotopic tumour-bearing HCC mouse 24 h after Fenestra LC administration, 10 days post-transplant. Sagittal, coronal and transverse planes are shown from left to right in the left panel. White dotted lines delineate the liver tumour. In the right panel, transverse plane of the MRI scan (top) confirms the demarcation of the tumour on Fenestra LC contrast-enhanced CT scans (bottom) (white arrowheads). MRI/CT intensity scales in greyscale show the minimum (bottom) and maximum (top) range. *N*=10 mice. Note: CT image is also used in K. (M) Representative longitudinal transverse CT scans, built from 1440 projections, of a 25 µl tumour-bearing orthotopic HCC mouse 24 h after a single administration of Fenestra LC (baseline, same day) and up to 18 days later. Black dotted lines delineate tumour boundaries. CT density scale indicates the minimum and maximum range. CT scans have been captured using the same window level. *N*=2 mice.

Alongside establishment of a suitable monofocal orthotopic liver cancer model, we evaluated the CT-based detection and delineation of the tumours. As the use of arterial and venous phase i.v. contrast is recommended for CT imaging of liver cancer (Chernyak et al., 2018), we first tested a time course of i.v. iodine-based (Omnipaque) contrast agent in tumour-bearing mice. CT imaging prior to and at several timepoints after i.v. contrast failed to detect the orthotopic liver tumours (Fig. 2A-C), consistent with previous reports in orthotopic models using other HCC cell lines (Zhou et al., 2023).

Next, we employed intraperitoneal (i.p.) administration of iodine (Omnipaque) contrast, with the aim of using hepatic surface irregularity to identify the orthotopic tumours. Although the liver outline was clearly delineated on CT imaging, this approach also failed to accurately identify the location or extent of the intrahepatic tumours (Fig. 2D-F). The failure of these CT imaging methods was confirmed by co-registration of CT and MRI images, which showed that tumours were visible on non-contrast enhanced MRI but not on CT (Fig. 2G,H).

We then employed a liver-specific cellular-based contrast agent, Fenestra LC, which is an iodine-based lipid emulsion, modelling chylomicron remnant uptake by hepatocytes, designed to be taken up specifically in the hepatic parenchyma. We found that i.v. injection of Fenestra LC resulted in enhancement of the liver parenchyma and clear delineation of non-enhancing tumours on CT images taken 10 days after 10 µl orthotopic transplantation (Fig. 2I-K). Co-registration of Fenestra LC contrast-enhanced CT and non-contrast enhanced MRI images confirmed accurate detection and delineation of the liver tumours (Fig. 2L). Furthermore, we were able to use non-invasive CT imaging to track the growth of untreated tumours for up to 18 days following a single injection of Fenestra LC contrast (Fig. 2M). This approach could therefore be used for evaluation of tumour growth in response to therapy over time *in vivo*, and to reduce the number of animals required for longitudinal studies.

### Tolerability of radiation delivery by the Small Animal Radiation Research Platform (SARRP) in wild-type mice

**Fig. 3.**
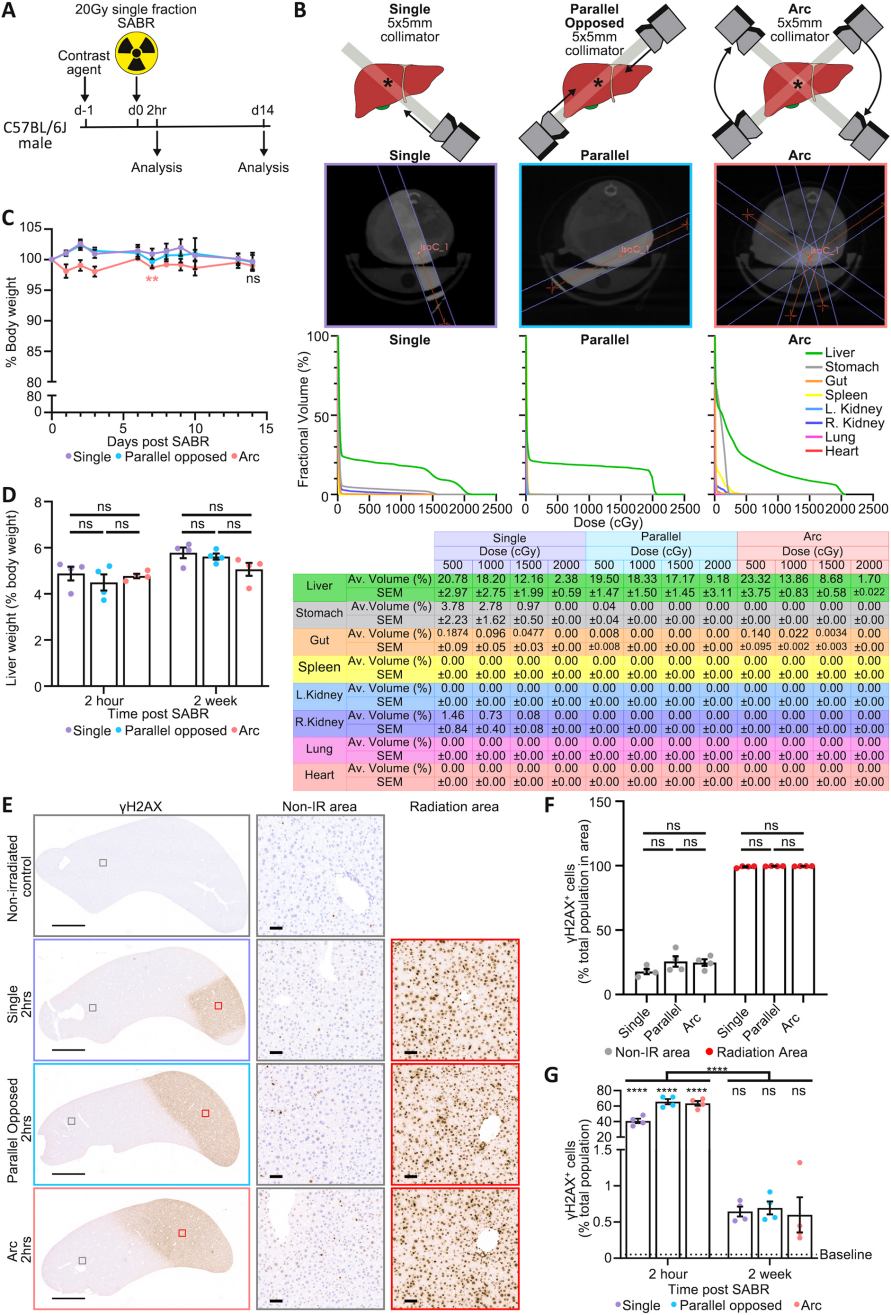
**CT-guided stereotactic liver irradiation in the healthy murine liver.** (A) Schematic of the *in vivo* hepatic irradiation toxicity study in wild-type mice. Male C57BL/6J mice were administered i.v. Fenestra LC contrast agent 24 h prior to receiving contrast-enhanced CT image-guided 20 Gy single-fraction stereotactic ablative radiotherapy (SABR), delivered via the Small Animal Radiation Research Platform (SARRP). The irradiation beam was delivered as a single, parallel opposed or dual arc configuration using a 5×5 mm collimator. Mice were sampled either 2 h or 2 weeks after receiving SABR. (B) Schematic and representative radiation plans on a 1440-projection CT scan in male C57BL/6J mice, showing the three different beam configurations utilised to deliver 20 Gy single-fraction SABR 24 h after receiving Fenestra LC to provide tissue contrast (*N*=4 mice per beam configuration). In these studies, radiation planning was designed to spare as much of the stomach and gut as possible. Respective dose-volume histograms (DVHs) and table showing the mean radiation dose received by the liver and organs-at-risk (OAR; stomach, gut, spleen, left and right kidneys, pancreas, lungs and heart) for each configuration (*N*=3 mice per beam configuration from the 2-h timepoint). (C) Percentage changes in body weight were calculated for male C57BL/6J mice immediately prior to (day 0) and for 14 days after receiving 20 Gy single-fraction SABR to the liver. *N*=4 mice per timepoint per beam configuration. Data shown as mean±s.e.m. Two-way ANOVA with Dunnett's multiple comparisons on paired data comparing body weights at each timepoint to baseline weight (day 0); ns, not significant *P*>0.05; **P=0.0061. (D) Liver weight to body weight ratios (%) calculated for male C57BL/6J mice 2 h and 2 weeks after receiving 20 Gy single-fraction SABR. *N*=4 mice per timepoint per beam configuration. Data shown as mean±s.e.m. Two-way ANOVA with Šídák's multiple comparisons test; ns, not significant *P*>0.05. (E) Representative images of immunohistochemistry (IHC) for γH2AX (brown) in livers in the radiation path and non-irradiated liver tissue control. Tissues were harvested from the male C57BL/6J mice 2 h after single-fraction 20 Gy therapy delivered using a 5×5 mm collimator. Inset images show γH2AX staining in areas of the liver within (radiation area) and outside (non-IR area) the radiation pathway. *N*=4 mice per cohort. Scale bars: 3 mm (main images); 50 µm (insets). (F) Quantification of γH2AX-positive cells within in the radiation path of each beam configuration and non-irradiated liver tissue of C57BL/6J mice 2 h post-irradiation. *N*=4 mice per cohort. Data shown as mean±s.e.m. Two-way ANOVA with Šídák's multiple comparisons test; ns, not significant *P*>0.05. (G) Quantification of γH2AX-positive cells within whole liver lobes of non-irradiated (baseline) and irradiated (2 h and 2 weeks after 20 Gy irradiation) C57BL/6J mice. *N*=4 mice per cohort. Data shown as mean±s.e.m. Two-way ANOVA with Dunnett's multiple comparisons comparing each radiation beam to non-irradiated controls; ns, not significant, *P*>0.05; ****P≤0.0001. Statistical comparisons between 2 h versus 2 weeks are reported as the interaction factor from two-way ANOVA.

**Fig. 4.**
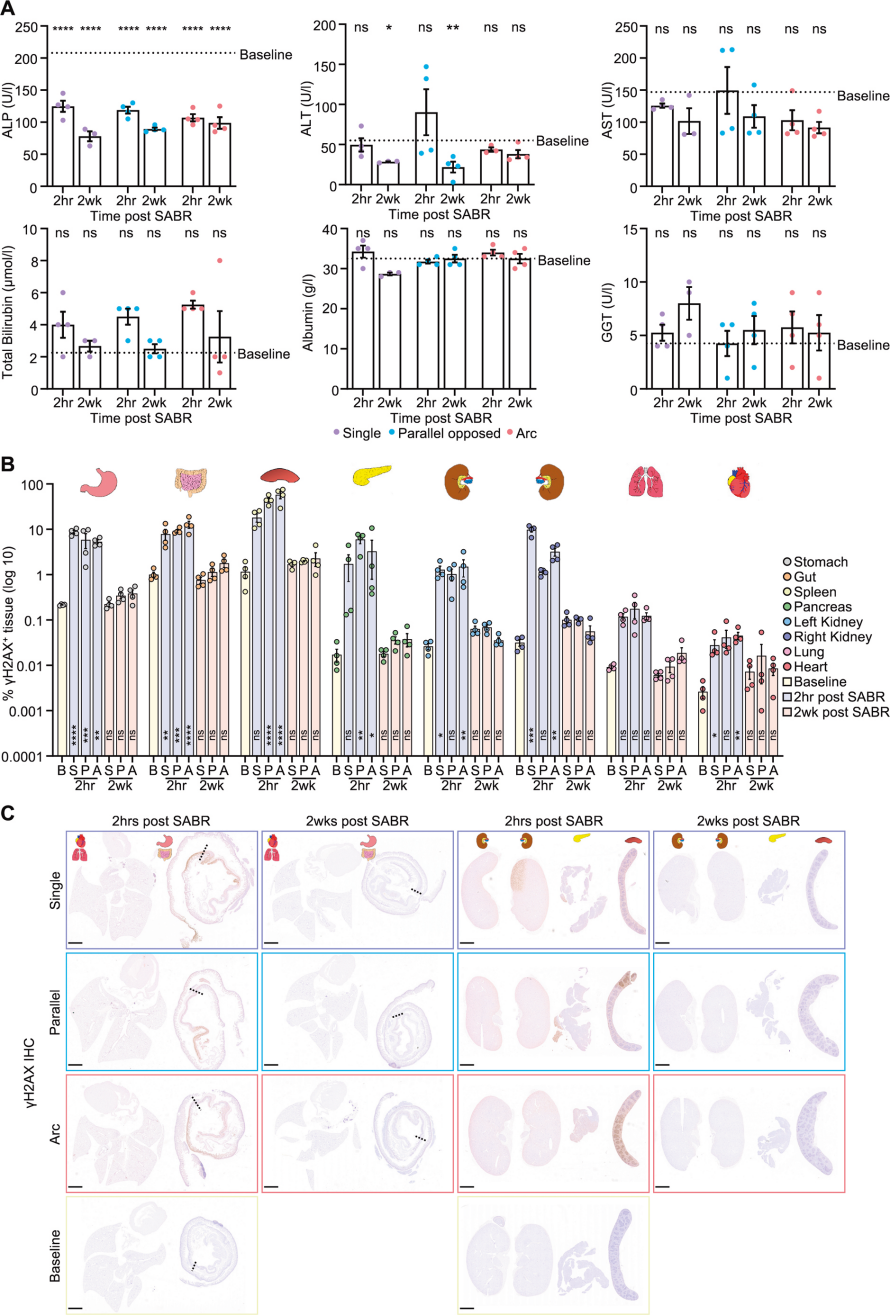
**Limited hepatic or systemic effects of stereotactic irradiation in the healthy liver.** (A) Plasma analysis of alkaline phosphatase (ALP), alanine transaminase (ALT), aspartate aminotransferase (AST), total bilirubin, albumin and gamma-glutamyl transferase (GGT) from wild-type C57BL/6J male mice 2 h and 2 weeks after receiving single-fraction 20 Gy SABR. *N*=3-4 mice per cohort. Statistical comparisons compare each radiation beam configuration at each timepoint post-SABR to non-irradiated baseline controls via one-way ANOVA with Holm-Šídák's multiple comparisons or Kruskal–Wallis test with Dunn's multiple comparison for parametric/non-parametric data, respectively. Data shown as mean±s.e.m. ns, not significant, *P*>0.05; *P≤0.05; **P≤0.01; ****P≤0.0001. Note: in some cohorts *N*=3 mice rather than *N*=4 owing to insufficient volume of plasma from each mouse to analyse all liver function tests. (B) Quantification of γH2AX positivity by tissue area in OAR (stomach, gut, spleen, pancreas, left and right kidneys, lung and heart) from male wild-type C57BL/6J mice treated with 20 Gy single-fraction SABR at 2 h (blue) and 2 weeks (red) compared to non-irradiated controls (baseline, yellow). B, baseline; S, single beam; P, parallel opposed beam; A, arc beam. *N*=4 mice per cohort. Data shown as mean±s.e.m. Two-tailed one-way ANOVA with Holm-Šídák's multiple comparisons (to baseline) or Kruskal–Wallis with Dunn's multiple comparison tests (to baseline) for parametric/non-parametric data, respectively. ns, not significant, *P*>0.05; *P≤0.05; **P≤0.01; ***P≤0.001; ****P≤0.0001. (C) Representative low-power microscopic images of each OAR from individual male wild-type C57BL/6J mice treated as in B. Scale bars: 2 mm; dotted lines represent the transition from stomach tissue to small intestinal tissue.

**Fig. 5.**
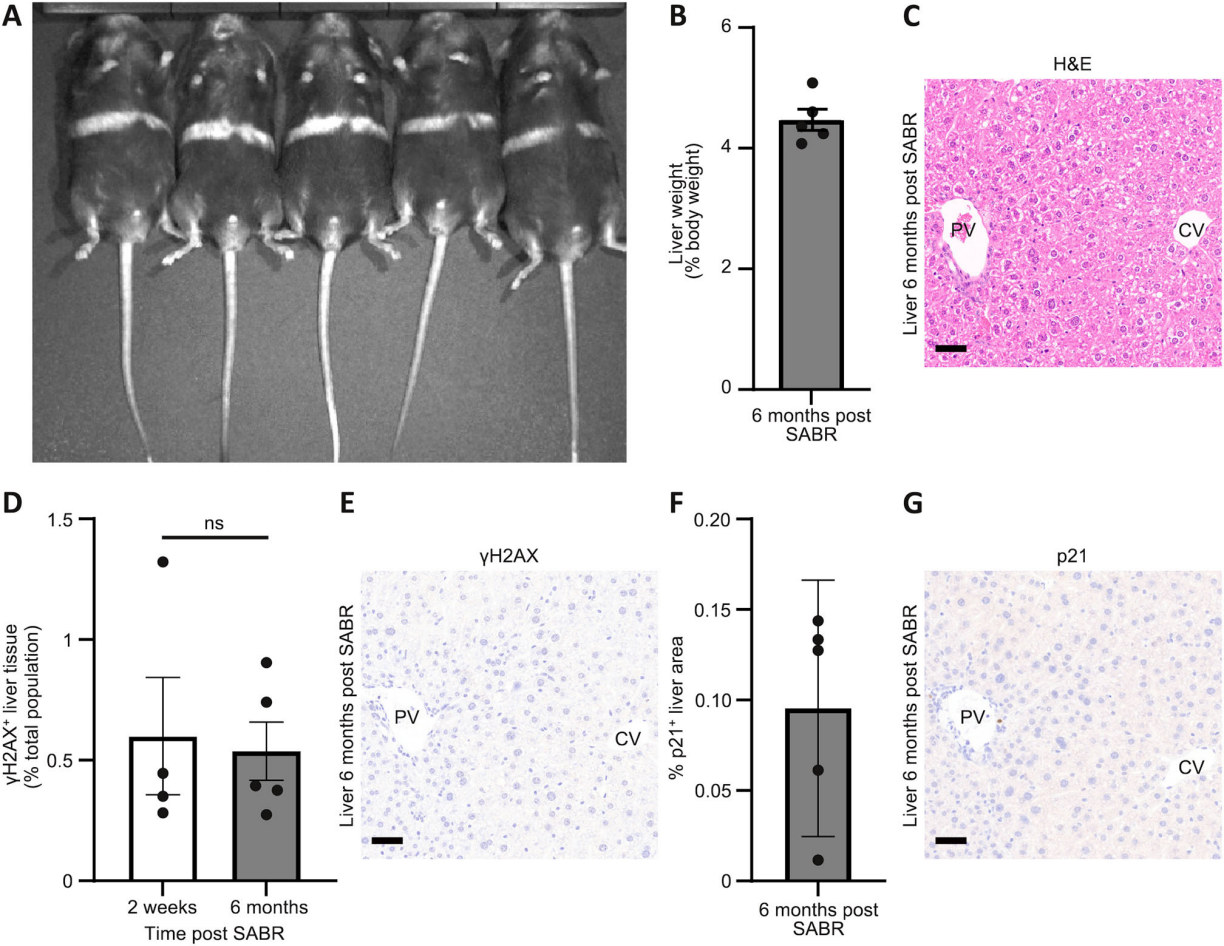
**Long-term health following stereotactic irradiation in the healthy liver.** (A) In Vivo Imaging System (IVIS)-acquired image of five wild-type C57BL/6J male mice 6 months after receiving single-fraction 20 Gy arc SABR. The previously black fur lost pigmentation along the arc radiation path (white band). *N*=5 mice as shown. (B) Liver weight to body weight ratios (%) were calculated for male wild-type C57BL/6J mice 6 months after receiving 20 Gy single-fraction arc SABR. Data shown as mean±s.e.m. Note that healthy liver weight/body weight ≈4.5%. *N*=5 mice. (C) Representative image of Haematoxylin and Eosin (H&E)-stained liver from a male wild-type C57BL/6J mouse 6 months after receiving 20 Gy single-fraction arc SABR. Scale bar: 50 μm. PV, portal vein; CV, central vein. Representative of *N*=5 mice. (D) Quantification of γH2AX-positive cells within the liver of male wild-type C57BL/6J mice 2 weeks or 6 months after receiving 20 Gy single-fraction arc SABR. *N*=5 mice at 6 months, *N*=4 mice at 2 weeks (note: the 2-week arc γH2AX positivity data are also used in Fig. 3G). Data shown as mean±s.e.m. Mann–Whitney test; ns, not significant, *P*>0.05. (E) Representative image of IHC for γH2AX (brown) in a liver from a male-wild type C57BL/6J mouse 6 months after receiving 20 Gy single-fraction arc SABR. Representative of *N*=5 mice. Scale bar: 50 µm. (F) Quantification of p21-positive tissue area within the liver of male wild-type C57BL/6J mice 6 months after receiving 20 Gy single-fraction arc SABR. *N*=5 mice. Data shown as mean±s.e.m. (G) Representative image of IHC for p21 (brown) in a liver from a male wild-type C57BL/6J mouse 6 months after receiving 20 Gy single-fraction arc SABR. Representative of *N*=5 mice. Scale bar: 50 µm.

The clinical application of SABR in HCC involves both fractionated and single-fraction approaches, with a lack of consensus on the optimum dose and scheduling. To maximise mouse welfare and simplify the experimental pipeline, we opted to employ a single fraction of 20 Gy. We first tested tolerability in non-tumour-bearing, wild-type C57BL/6J male mice. Guided by Fenestra LC contrast-enhanced CT imaging, we delivered 20 Gy with a 5 mm collimator to an isocentre positioned within the left lobe of the liver, the site of tumour implantation in the orthotopic model. Radiation was delivered using one each of three different beam configurations (single, parallel opposed or dual arc), and mouse health was monitored for 2 weeks following irradiation (Fig. 3A,B). Dose-volume histograms (DVHs) derived from the radiotherapy treatment plans indicated minimal irradiation of local organs or tissues (Fig. 3B). No adverse clinical signs were observed; generally, mice maintained their body weight for up to 14 days (Fig. 3C), and there was no change in liver mass (Fig. 3D). No differences were observed between the three different beam configurations. Microscopic analysis of the livers and other organs was then performed to assess targeted and off-target DNA damage effects immediately following (2 h) and after recovery from the procedure (2 weeks). Here, we observed clearly demarcated γH2AX expression within the irradiated liver indicative of radiation-induced DNA damage within the targeted area, with different geometrical areas affected according to the beam arrangement, as expected (Fig. 3E). More than 98% of cells within targeted areas expressed γH2AX (Fig. 3F) at 2 h post-irradiation. Assessing the whole liver, γH2AX expression fell dramatically over the recovery period, with minimal staining at the 2-week timepoint (Fig. 3G). Biochemical measurement of serum liver function tests revealed no demonstrable overt impact of irradiation on the healthy liver (Fig. 4A) at either 2-h or 2-week timepoints. Examining effects of irradiation on local organs, known as ‘organs-at-risk’ (OAR), we observed ‘off-target’ expression of γH2AX in regions of the stomach, small intestine and spleen, which lie anatomically adjacent to the irradiated liver. The proportion of cells displaying evidence of DNA damage in these organs was low (typically <10% but up to 60% in spleen) and in all cases had returned to baseline at week 2 (Fig. 4B,C), without evidence of adverse clinical signs. To ensure that this CT-targeted SABR approach was well tolerated, we monitored mice for 6 months following irradiation. All mice remained healthy, with the only clinical sign being a band of hair discolouration corresponding to the radiation beams (Fig. 5A). Liver weight to body weight ratios remained within normal ranges (Fig. 5B); livers remained normal histologically (Fig. 5C), and there was no long-term expression of markers of DNA damage (γH2AX) or senescence (p21; also known as CDKN1A) (Fig. 5D-G). In summary, targeted SABR in the liver, utilising a clinically relevant radiotherapy dose, was well tolerated in healthy mice.

### Targeted irradiation of liver tumours in orthotopic mice

**Fig. 6.**
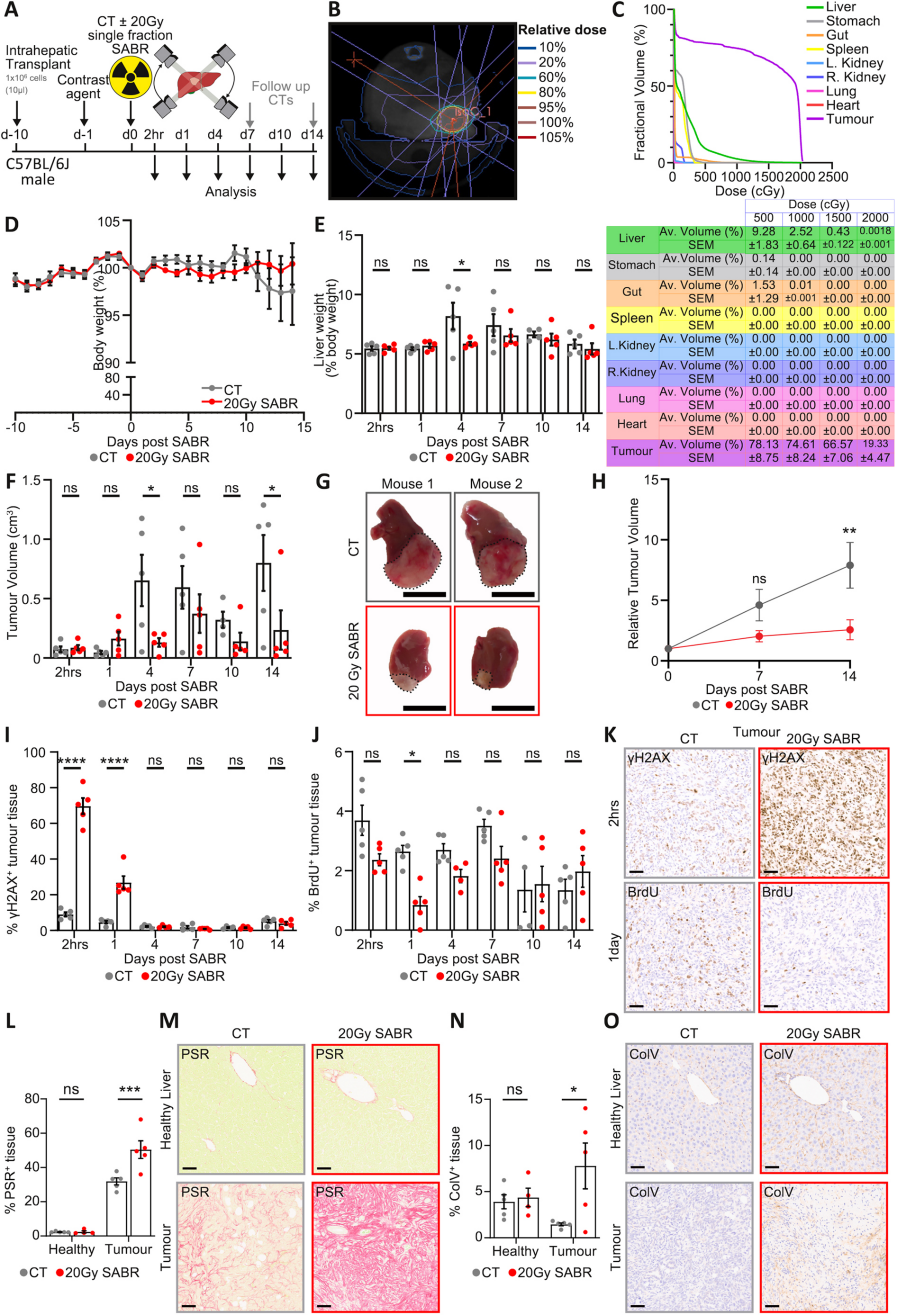
**CT-guided stereotactic tumour irradiation in the HCC orthotopic model.** (A) Timeline schematic of the orthotopic HCC model with hepatic irradiation. Male C57BL/6J mice received an intrahepatic injection of one million single Hep53.4 HCC cells into a lobe of the liver in 10 µl Matrigel. All mice received i.v. Fenestra LC contrast agent 24 h prior to receiving either CT-guided 20 Gy single-fraction arc SABR to the tumour isocentre or a CT scan only. Mice were sampled at various times as shown post-SABR to assess tumour growth; d, days post-irradiation. Follow-up CT imaging was conducted at day 7 and day 14 post-SABR. (B) Representative radiation plan on a Fenestra LC contrast-enhanced 1440-projection CT scan in HCC tumour-bearing C57BL/6J mice, showing an arc beam utilised to deliver 20 Gy single-fraction SABR (representative of *N*=5 mice per cohort). In these studies, radiation planning was designed to spare as much of the stomach and gut as possible. (C) DVHs and table showing the volume (%) of tissue receiving a radiation dose in the tumour, liver and OAR (stomach, gut, spleen, left and right kidneys, pancreas, lungs and heart) (*N*=5 mice from the 2-h timepoint). (D) Percentage changes in mouse body weight were calculated for male orthotopic HCC tumour-bearing mice before and after transplant (day 10) and during and after SABR therapy. *N*≥5 mice at each timepoint per therapy. Data shown as mean±s.e.m. Two-way ANOVA with Dunnett's multiple comparisons test on paired data comparing body weights from CT only and 20 Gy SABR at each timepoint; no statistical significance (*P*>0.05) was detected at any timepoint. (E) Liver weight to body weight ratios calculated for male tumour-bearing mice at different timepoints after receiving 20 Gy single-fraction arc SABR and compared to non-irradiated tumour-bearing controls. Data shown as mean±s.e.m. *N*=5 mice for all cohorts except at day 10, when the CT arm had *N*=4 mice. Two-way ANOVA with Šídák's multiple comparisons test; ns, not significant, *P*>0.05; *P=0.0173 for the day 4 comparison. (F) Quantification of tumour burden (cm^3^) for the orthotopic HCC mice at each timepoint post-SABR compared to non-irradiated tumour-bearing controls. Tumour volumes were calculated from calliper measurements at the time of cull. Data shown as mean±s.e.m. *N*=5 mice for all cohorts except at day 10, when the CT arm had *N*=4 mice. Two-way ANOVA with Šídák's multiple comparisons test; ns, not significant, *P*>0.05; *P≤0.05. (G) Representative images of liver lobes from the orthotopic HCC model 2 weeks post-20 Gy single-fraction arc SABR or CT only, Representative of *N*=5 mice per cohort. Dotted black lines indicate the orthotopic tumour boundary. Scale bars: 1 cm. (H) Relative tumour volume from monitoring of longitudinal Fenestra LC contrast-enhanced CT scans of tumour-bearing HCC mice left unirradiated or exposed to 20 Gy SABR during CT scan acquisition on day 0. Data shown as mean±s.e.m. *N*=5 mice for CT and *N*=4 mice for SABR. Two-way ANOVA with Šídák's multiple comparisons test comparing SABR to CT tumour volumes at each timepoint; ns, not significant, *P*>0.05 at day 7; **P=0.0087 at day 14. (I) Quantification of γH2AX positivity by tissue area in tumours from male orthotopic HCC mice at each timepoint post-SABR compared to non-irradiated tumour-bearing controls. *N*=5 mice for all cohorts except at days 4 (SABR arm) and 10 (CT arm), each of which had *N*=4 mice. Data shown as mean±s.e.m. Two-way ANOVA with Šídák's multiple comparisons test comparing SABR to CT at each timepoint; ns, not significant, *P*>0.05; ****P≤0.0001. (J) Quantification of BrdU positivity by tissue area in tumours from male orthotopic HCC mice at each timepoint post-SABR compared to non-irradiated tumour-bearing controls. *N*=5 mice for all cohorts except at days 4 (SABR arm) and 10 (CT arm), each of which had *N*=4 mice. Data shown as mean±s.e.m. Two-way ANOVA with Šídák's multiple comparisons test comparing SABR to CT at each timepoint; ns, not significant, *P*>0.05; *P=0.0143. (K) Representative images of IHC for γH2AX and BrdU (brown) in an orthotopic HCC tumour 2 h (γH2AX) and 1 day (BrdU) after receiving 20 Gy single-fraction arc SABR and a non-irradiated tumour-bearing control (received CT scan only). Each representative of *N*=5 mice. Scale bars: 50 µm. (L) Quantification of Picrosirius Red (PSR) positivity by tissue area in healthy liver and tumours from male orthotopic HCC mice at 14 days post-SABR compared to non-irradiated (received CT scan only) tumour-bearing controls. *N*=5 mice for all cohorts except for healthy liver of mice receiving SABR to the tumours, for which *N*=4 mice owing to a lack of healthy tissue in the histology section. Data shown as mean±s.e.m. Two-way ANOVA with Šídák's multiple comparisons test comparing SABR to CT in either the non-irradiated healthy liver tissue or tumour; ns, not significant, *P*>0.05; ***P=0.0008. (M) Representative images of PSR staining (pink) in healthy liver and tumours of an orthotopic HCC mouse 14 days after receiving 20 Gy single-fraction arc SABR and a non-irradiated tumour-bearing control (received CT scan only). Representative of *N*≥4 mice. Scale bars: 50 µm. (N) Quantification of collagen V (ColV) positivity by tissue area in healthy liver and tumours from male orthotopic HCC mice at 14 days post-SABR compared to non-irradiated (received CT scan only) tumour-bearing controls. *N*=5 mice for all cohorts except for healthy liver of mice receiving SABR to the tumours, for which *N*=4 mice owing to a lack of healthy tissue in the histology section. Data shown as mean±s.e.m. Two-way ANOVA with Šídák's multiple comparisons test comparing SABR to CT in either the non-irradiated healthy liver tissue or tumour; ns, not significant, *P*>0.05; *P=0.0121. (O) Representative images of ColV staining (brown) in healthy liver and tumours of an orthotopic HCC mouse 14 days after receiving 20 Gy single-fraction arc SABR and a non-irradiated tumour-bearing control (received CT scan only). Representative of *N*≥4 mice. Scale bars: 50 µm.

Next, we integrated our highly targeted SABR strategy with contrast-enhanced CT-based delineation of orthotopic HCC tumours. Given the clinical relevance of arc radiotherapy, and our previous demonstration of tolerability, we utilised the dual arc configuration in subsequent cancer therapy studies. Because tumours were detected in all mice 10 days after 10 µl orthotopic transplantation, we sampled mice at prespecified timepoints after liver tumour SABR administered 10 days post-transplantation (Fig. 6A). Each mouse was irradiated with a focused tumour-based collimator of 3-7 mm (depending upon individualised imaging), delivering 20 Gy to the isocentre (Fig. 6B). DVHs demonstrate focused irradiation of the tumour, with low doses to up to 50% of the surrounding liver and minimal irradiation of surrounding OAR (Fig. 6C). All mice recovered well from the irradiation procedure, maintaining weight over the following 2 weeks (Fig. 6D). Liver and tumour tissues were harvested 2 h and 1, 4, 7, 10 and 14 days after SABR. Liver weights were stable (Fig. 6E), but mean tumour volumes were lower on days 4 and 14 after irradiation compared with CT-only control, with trends towards reduced volume at 7 and 10 days (Fig. 6F-H). Microscopic examination of tumours demonstrated high levels of γH2AX staining at 2 h and moderate levels at 1 day, and a transient reduction in tumour cell proliferation was indicated by reduced 5-bromo-2'-deoxyuridine (BrdU) staining 1 day post-irradiation (Fig. 6I-K). Tumour irradiation was associated with increased fibrosis within tumours but not in normal liver, as indicated by elevated Picrosirius Red (Fig. 6L,M) and collagen V (ColV; also known as COL5) staining within tumour tissue (Fig. 6N,O).

**Fig. 7.**
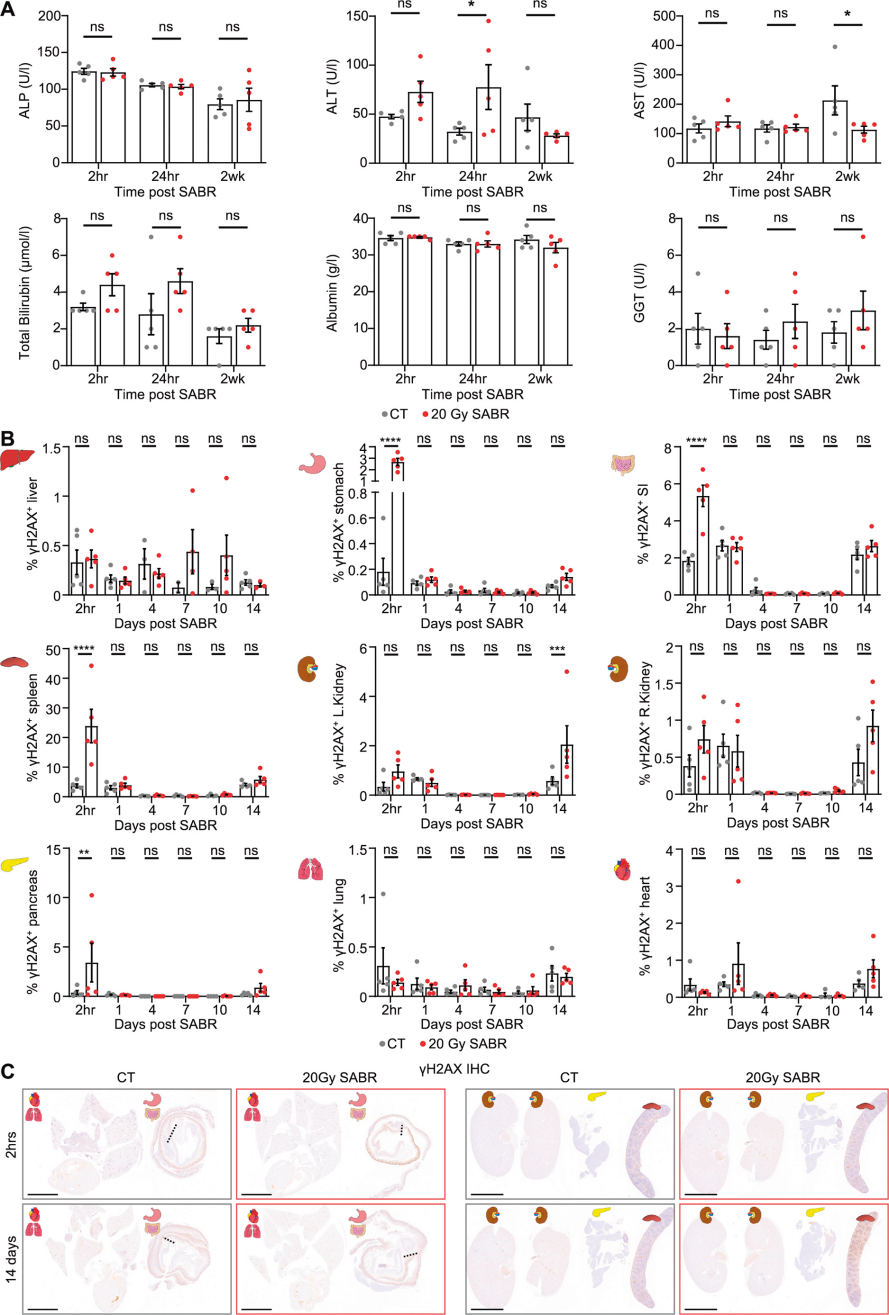
**Limited hepatic or systemic effects following stereotactic irradiation of liver tumours.** (A) Plasma analysis of ALP, ALT, AST, total bilirubin, albumin and GGT from male orthotopic HCC mice 2 h, 24 h and 2 weeks after receiving single-fraction 20 Gy arc SABR. *N*=5 mice per cohort. Statistical comparisons compare each SABR to non-irradiated tumour controls (received CT scan only) at each timepoint via two-way ANOVA with Šídák's multiple comparisons test. Data shown as mean±s.e.m. ns, not significant, *P*>0.05; *P≤0.05. (B) Quantification of γH2AX positivity by tissue area in healthy liver tissue and OAR (stomach, gut, spleen, pancreas, left and right kidneys, lungs and heart) from male orthotopic HCC mice at different timepoints after receiving single-fraction 20 Gy arc SABR to tumours compared to non-irradiated tumour-bearing controls. *N*=5/5, *N*=5/5, *N*=3/5, *N*=2/4, *N*=3/5 and *N*=5/3 mice for CT/SABR at 2 h, 1 day, 4 days, 7 days 10 days and 14 days post-treatment, respectively, in liver; *N*=5 mice for all cohorts except at day 10, when the CT arm has *N*=4 mice, in OAR. *N* number is low in some cohorts owing to limited healthy tissue in some histology sections. Data shown as mean±s.e.m. Two-way ANOVA with Šídák's multiple comparisons test comparing SABR to CT at each timepoint; ns, not significant, *P*>0.05; **P≤0.01; ***P≤0.001; ****P≤0.0001. (C) Representative low-power microscopic images of each OAR from individual male orthotopic HCC mice treated with 20 Gy single-fraction SABR at 2 h and 14 days compared to non-irradiated controls. Representative of *N*=5 mice per cohort. Scale bars: 4 mm; dotted lines represent the transition from stomach tissue to small intestinal tissue.

Liver biochemistry showed only minimal changes following tumour irradiation, with a mild elevation in serum transaminases [alanine transaminase (ALT) at 1 day, aspartate transaminase (AST) at 2 weeks], indicative of mild hepatocellular or tumour injury (Fig. 7A). The remaining liver, unlike the irradiated tumour, showed very little evidence of DNA damage at any timepoint examined (Fig. 7B). There was, as predicted, mild transient elevation of γH2AX in the OAR (Fig. 7B,C), consistent with the irradiation received by these organs (Fig. 6C) and peaking within 1 day of irradiation. In summary, we describe a reproducible model of stereotactic liver tumour irradiation, with evidence of a well-tolerated SABR strategy and on-target antitumour effects.

## DISCUSSION

Here, we describe the combination of several elements that provide a highly penetrant and consistent HCC tumour model, which provides monocentric tumours that can be delineated using contrast-enhanced CT imaging and then subjected to image-guided SABR, modelling the SABR procedure performed in patients with HCC. We demonstrate that this approach delivers clinically relevant high-dose radiotherapy to the tumour, which is well tolerated with detectable but clinically insignificant off-target effects. Tumour irradiation in the model has biologically and clinically meaningful impact, reducing tumour volume and inducing DNA damage in residual tumours. The model also permits longitudinal non-invasive, volumetric imaging to track effects on the primary tumour. Furthermore, this model platform enables integration and evaluation of multimodal therapy for HCC.

SABR therapy for HCC is included in numerous international HCC therapy guidelines (Ducreux et al., 2023; Singal et al., 2023; Sangro et al., 2025; European Association for the Study of the Liver, 2018). However, there are major unanswered questions about optimisation of this treatment in terms of target volume, dose, fractionation schedule and how it could be most effectively combined with other forms of therapy. For example, immunotherapy has been shown to enhance radiotherapeutic effects in other diseases, and the changes in the immune environment that occur in HCC make this an attractive opportunity to explore experimentally for clinical translation (Bettinger et al., 2019). In HCC, systemic therapy options include immunotherapy and tyrosine kinase inhibition. Immunotherapy combinations are generally considered the first line of systemic therapy currently, but response rates remain poor (Sangro et al., 2025). Attempts to identify subpopulations of patients with HCC that are most likely to respond to immunotherapy have failed thus far. There remain opportunities to sensitise the tumour microenvironment to immunomodulatory therapies, including the use of SABR. Our preclinical platform for SABR modelling in the mouse offers the opportunity to test such therapeutic combinations. For example, modulation of responses to immunotherapy in the orthotopic Hep53.4 model have already been described (Leslie et al., 2022). It remains to be tested whether this can be further manipulated by addition of SABR and, if so, how this can be optimised. Numerous other therapeutic strategies could be integrated into this system, for example combination with tyrosine kinase inhibition, altering the background liver disease [e.g. through the introduction of metabolic dysfunction-associated steatotic liver disease (MASLD)], manipulating the Hep53.4 line (e.g. CRISPR editing) or the transplantation of alternative HCC lines in a similar system. All would require appropriate optimisation of the pipeline described here, including formation of consistent monofocal tumours, their visualisation and monitoring post-SABR.

We have chosen the Hep53.4 cell line for a variety of reasons. It has been successfully employed by us and others for transplantation into immunocompetent C57BL/6 mice (Leslie et al., 2022, 2023; Barcena-Varela et al., 2024). The cell line originates from a tumour derived from a C57BL/6 mouse and, despite its wide mutational burden, engrafts consistently, creating an immunologically active tumour immune microenvironment (Müller et al., 2025; Leslie et al., 2022). The orthotopic tumours are partially responsive to immunotherapy, making it an attractive target for the multimodal therapy approaches discussed above. The addition of alternative cell lines, e.g. Hep1-6 (a cell line from a spontaneous HCC), could alternatively be optimised to create a broader suite of orthotopic models for SABR. Similarly, our recent description of a broad range of HCC genetically engineered mouse models (GEMMs) and organoid lines (Müller et al., 2025) provides further opportunities for organoid-derived orthotopic models recapitulating a wide range of HCC heterogeneity into this SABR platform. These may facilitate study of radiotherapy responses in immune-active and immune-evasive disease or cells with intact versus defective cell cycle checkpoints (e.g. p53 deleted), for example.

In addition to modifications of the tumour itself, this model platform also lends itself to modification to the host. Host GEMMs would allow alternations to the liver parenchyma (Gay et al., 2019) or investigate non-parenchymal populations (Leslie et al., 2022). Equally, the background liver could be modified with chronic injury, such as fibrosis or steatosis. Notably, modelling responses in MASLD has already been shown to affect therapeutic responses in both the orthotopic setting (Leslie et al., 2022) and in other HCC models (Pfister et al., 2021).

A further opportunity arising from this platform is the ability to study abscopal effects of tumour-targeted therapy such as SABR. Abscopal effects (those of locally restricted therapy both on that location and distant tumours) have been described in a variety of tumours following irradiation (Craig et al., 2021) including HCC (Ohba et al., 1998; Nakabori et al., 2024). Mechanistic study of the interactive effects of multimodal therapy upon the tumour and metastatic disease, as occurs in this Hep53.4 orthotopic model and more recently described HCC GEMMs, is tractable using the preclinical model system we describe here. Intriguingly, we observe a delayed off-target renal phenotype following liver SABR (Fig. 7B) that may mirror the inter-organ transmission of senescence, which has recently been described from the liver to the kidney epithelium (Kiourtis et al., 2024).

There are limitations to our platform. The models described here do not reflect the chronicity of human HCC development, nor do they occur on a background of liver cirrhosis as is typical in patients with HCC. As discussed previously, we have not yet validated the effects of fractionated SABR therapy in the model, although this is likely to be technically possible, particularly given the long-lasting contrast enhancement of the liver, facilitating longitudinal tumour delineation, observed with our imaging approach. Although we are able to model a clinically relevant SABR arc delivery approach, the small size of the mouse means that there is a very small distance between the liver tumour and surrounding OAR, resulting in greater off-target irradiation of these organs compared with the human. Taken as a whole, however, we believe that this platform is an important advancement over those developed previously (Kabarriti et al., 2019; Zhou et al., 2023). Our findings are consistent with the first report of SABR delivered to the liver, which demonstrated good tolerability in healthy tumour-free mice (Kabarriti et al., 2019). In a more recent report, describing imaging of and delivery of SABR to tumour-bearing livers in the mouse (Zhou et al., 2023), the authors conceded that they were unable to target the tumour directly owing to difficulties in tumour visualisation and image registration for therapeutic planning. Our approach has overcome this significant challenge and allows accurate targeting of high-dose radiotherapy to the tumour. Although we see biological effects of targeted SABR upon tumours, tumours persist and continue to proliferate. This mimics the radioresistant nature of some HCCs and provides opportunities to evaluate combination treatment approaches. Further optimisation of dose and fractionation will also be important to study. From our studies, based upon power calculations with 80% power to detect 40% treatment effect with alpha 0.05, we would recommend using biological replicates of at least six to ten animals per treatment arm in future studies to detect changes in radiological tumour volume (in non-irradiated tumours *n*=6/8 at days 7/14, respectively, and in irradiated tumours *n*=5/10 at days 7/14, respectively). Overall, however, our demonstration of a tractable model platform for integrating SABR therapy into preclinical HCC modelling offers a platform on which to unlock mechanistic insights into the role of SABR in liver cancer.

## MATERIALS AND METHODS

### Hep53.4 cell culture

Hep53.4 cells (RRID:CVCL_5765; derived from a C57BL/6J primary HCC) were purchased from Cytion (LOT-L230232R) and were routinely cultured in Dubecco's modified Eagle medium (DMEM) with 4.5 g/l glucose and sodium pyruvate (Thermo Fisher Scientific, 10313021), supplemented with 10% fetal bovine serum (FBS; Gibco, 10270106) and 1% Gibco GlutaMAX™ Supplement (Thermo Fisher Scientific, 35050038). Cells were maintained in T175 flasks and incubated at 37˚C and 5% CO_2_. Medium was changed twice a week, and cells were passaged using Tryple Express (Thermo Fisher Scientific, 12604013) when they reached ∼80% confluency. The number of viable cells was determined using 0.4% Trypan Blue stain (Gibco, 15250-061) on a DeNovix Fluorescence cell counter machine. Cells were seeded at their desired density in new flasks or plates (*in vitro*) or in Corning Matrigel (*in vivo*). Mycoplasma testing was routinely performed in each instance prior to cell transplantation (*in vivo*) or cell seeding (*in vitro*) in house (protocol available upon request), and cultures always tested negative for Mycoplasma contamination.

### *In vitro* clonogenic assay

To determine whether Hep53.4 cells are radiosensitive, Hep53.4 cells were seeded in six-well plates at 500 or 1000 cells per well for 0-4 Gy and 6-10 Gy, respectively. Twenty-four hours after cell seeding, plates were either sham irradiated (0 Gy) or irradiated at 2, 4, 6, 8 or 10 Gy at room temperature using a RS225 System (Xstrahl, GS040) with 195 kV/15 mA X-rays producing a dose rate of 1.39 Gy/min. Plates were then incubated for 2 weeks at 37°C in 5% CO_2_, without media changes prior to fixation with ice-cold 50:50 acetone:methanol and staining with 0.5% Crystal Violet (Sigma-Aldrich, HT90132-1L). Photomicrographs were taken using a Nikon camera (D5300+AF-P 18-55VR Digital SLR Camera & Lens Kit) and unaware of experimental group (picture identifiers were anonymised by another researcher), and visible colonies were counted manually using FIJI ImageJ (v2.14.0) (Schindelin et al., 2012). The average surviving fraction for the Hep53.4 clonogenic survival assay was taken across four experimental repeats (different passages of cells all under passage 18), each with six technical replicates. Clonogenic survival data were fitted using the linear quadratic model. Note: a fifth experimental repeat was excluded from the analysis owing to clumping together of colonies (at 0, 2 and 4 Gy), preventing accurate quantification of surviving clones.

### Orthotopic transplant

All mouse experiments were performed in accordance with a UK Home Office project licence (PP0604995; protocol numbers 3 and 8), in accordance with Home Office guidelines, and were subject to review by the animal welfare and ethical review board of the University of Glasgow. To minimise pain, suffering and distress to the animals, single-use needles and non-adverse handling techniques were used throughout. The mice used in this study were male C57BL/6J (commercially bought from Charles River Laboratories, UK). Mice were housed in a specific pathogen-free environment and kept under standard conditions with a 12 h day/night cycle with unrestricted access to food and water *ad libitum*. Environmental enrichments, in the form of bedding, plastic tunnels and chew sticks, were added to all cages. Plastic tunnels were removed from cages for the first 7-10 days after orthotopic transplant to avoid catching/tearing of the skin clips.

Age- and batch-matched commercially bought C57BL/6J male mice were given a minimum 1-week acclimatisation period before orthotopic intrahepatic injection between 8 and 13 weeks of age. All mice were administered 150 µl subcutaneous supportive fluids (PBS) in the flank and analgesia prior to surgery [5 mg/kg Carprofen (Rimadyl) in the drinking water from 24 h before surgery and for a further 48 h after surgery], and 0.03 mg/ml subcutaneous administration of Buprenorphine (Vetergesic) in the scruff. Mice were anaesthetised using sevoflurane (with O_2_) in a sterile surgical field. A median laparotomy was performed followed by expression of the left lateral liver lobe. One million Hep53.4 cells in 10 or 25 µl, as stated, of Corning Matrigel were injected into the left lobe of the liver using a Hamilton syringe. Sterile surgical gauze was placed over the injection site to prevent blood loss and leakage of cells. The liver was gently placed back inside the abdomen, and the muscle layer was closed with a continuous vicryl suture (Ethicon). A local anaesthetic [2.5 mg/ml Bupivacane (Marcaine)] was topically administered to the sutured wound prior to skin clipping. Hydrogel H_2_O and diet gel packs (Tecniplast) were added to the cage during recovery. Mice were placed in a warmed recovery cage to assist recovery for up to 4 h. Surgical clips were removed between 7 and 10 days post-transplantation. All mice were health checked daily and were housed in the same location.

### Dissection and scoring of tumour burden

All mice were administered BrdU (250 µl; GE Healthcare) via i.p. injection 2 h prior to culling. Mice were humanely culled at timepoints by rising concentrations of CO_2_ inhalation followed by cervical dislocation. Blood was collected by cardiac puncture and placed in lithium heparin collection tubes (Sarstedt). Following dissection, whole livers were weighed then further dissected into individual liver lobes, and visible macroscopic liver tumours were scored using digital callipers in either two (<1 cm) or three dimensions (≥1 cm) for calculating tumour volume (length/2×height/2×breath/2×4/3π). The liver/tumour and OAR were fixed in 10% neutral buffered formalin (in PBS) for 24 h.

### Liver biochemistry

Plasma samples were prepared following blood collection in heparin tubes (Sarstedt), centrifugation (5000 ***g*** for 5 min) and plasma separation, and stored at −80°C until required. Plasma samples were analysed for AST, ALT, alkaline phosphatase (ALP), total bilirubin, gamma-glutamyl transferase (GGT) and albumin using the Dimension Expand chemistry system (Siemens Healthcare Diagnostics), following International Federation of Clinical Chemistry-approved methods.

### CT imaging and contrast agent administration

The livers were imaged by cone beam CT, which is an in-built function of the SARRP (Xstrahl). CT parameters were 80 kV and 0.8 mA, and 180 or 1440 projections were acquired to reconstruct images of the mice. CT imaging dose at 1440 projections has been calculated to be 6.22 cGy. Animals were anaesthetised throughout the procedure with a mixture of isoflurane and medical air. Following CT acquisition or irradiation, mice were monitored during anaesthesia recovery in prewarmed cages.

Contrast agents were used to enhance the CT images. Omnipaque™ (Iohexol; GE Healthcare) was administered as either an i.v. or i.p. (as stated) injection in the 25 µl orthotopic HCC mouse model on the day of CT imaging, and Fenestra LC (MediLumine Inc., Montreal, Canada) as an i.v. injection in the 10 µl orthotopic HCC mouse model 9 days post-transplant. For i.v. administration of Omnipaque, 150 µl was administered (diluted 1:1 in PBS. For i.p. administration of Omnipaque, 200 µl was administered (diluted 1:1 in sterile water). For Fenestra LC dosing, mice first received 200 µl PBS via subcutaneous injection into the scruff as per the manufacturer's recommendation for better contrast agent tolerability, followed by i.v. injection of Fenestra LC based on body weight (10 µl/g). Fenestra LC was dosed ∼24 h prior to CT and/or SABR therapy (9 days post-transplant). Fenestra LC was ultimately used as the contrast of choice as it delineated the area of tumour within the liver. Fenestra LC shows prolonged contrast enhancement over time; therefore, multiple imaging sessions can be performed from a single administration for the duration of the studies.

Following irradiation/sham, mice were imaged weekly to track tumour progression again with a 1440 projection CT scan at 80 kV and 0.8 mA. Volume of tumour could also be tracked over multiple imaging sessions by contouring using VivoQuant 4.0 (Invicro, USA) imaging software.

### SARRP radiotherapy

Anaesthetised mice (wild-type and tumour-bearing) were irradiated with 20 Gy on day 10 after orthotopic implantation of HCC cells, using the SARRP. Using Muriplan, the SARRP's bespoke planning software, the CT images are utilised to select an area for targeted beam isocentre and to plan the irradiation to that isocentre. The SARRP then delivers a 220 kVp, 13 mA X-ray beam with a dose rate of ∼280 cGy/min to the mice. Treatment was delivered using various beam arrangements: single-beam delivery, parallel opposed beams or dual arc arrangement to the tumour isocentre. Collimator size for wild-type mice was 5×5 mm; for tumour-bearing mice, the collimator was guided by the size of the tumour imaged (e.g. 3×3 mm, 5×5 mm). Mock irradiated animals received a 1440 projection CT scan at 80 kV and 0.8 mA.

### Dose assessment by DVH

Tumour and OAR (e.g. liver, stomach and gut) were contoured using Muriplan's in-built module. Plans were evaluated by using the volumes to produce DVHs, allowing assessment of dose to tumour and dose to OAR per area for each dosing plan.

### MRI

MRI scans were performed in wild-type and liver tumour-bearing mice using a nanoScan^®^ 1T PET/MRI scanner (Mediso Imaging Systems, Hungary). Mice were maintained under inhaled isoflurane anaesthesia via nose cone (induction, 5% v/v; maintenance, 1.5-2.0% v/v) in 95% oxygen throughout MRI, while body temperature was maintained by a flow of heated air, and respiratory rate was monitored by a respiratory pressure pad sensor through the integrated ‘Mediso mouse monitoring system’, with adjustments in respiration made by regulating depth of anaesthesia by adjusting the concentration of isoflurane. All mice were scanned by using designated settings and imaging parameters in the ‘head-first-prone’ position. After preliminary scout images, whole-body T1-weighted gradient echo 3D axial MRI sequences were acquired. These images were acquired using a slice thickness of 0.6 mm to ensure complete coverage of the mice. The MRI parameters were as follows: repetition time (TR), 13.6 ms; echo time (TE), 2.2 ms; flip angle, 15°; and field of view, 36.0 mm×36.0 mm. Scans were evaluated by manually drawing the volume-of-interest (VOI) around the tumour region in MRI scans through visual inspection. Distinct VOIs were delineated for each mouse's scan to account for mouse positioning on the scanner and tumour size.

### CT-MRI co-registration

Co-registration of MRI and CT imaging was performed in a two-stage process using VivoQuant 4.0 (Invicro) software. A rigid translation to the scan was initially performed on the scans, a process involving adjusting the images for precise alignment without rotation. Following this initial alignment, co-registration was checked manually, and small adjustments to improve registration were applied where necessary. Finally, co-registered scans from VivoQuant were exported, in DICOM format, for further analysis.

### In Vivo Imaging System (IVIS) imaging

Mice were anesthetised with a mixture of isoflurane and medical air and placed on the imaging stage of the IVIS (Xenogen, Alameda, CA, USA) in the supine position (on their backs) before imaging.

### Histology

Dissected mouse tissue was fixed overnight in 10% neutral buffered formalin prior to transfer to 70% ethanol. Tissue was then processed with a standard overnight histology tissue-processing cycle before being orientated and embedded in histology wax. Immunohistochemistry (IHC), Haematoxylin and Eosin (H&E) and Picrosirius Red (PSR) staining took place on 4 µm formalin-fixed paraffin-embedded (FFPE) sections, after heating at 60°C for 2 h.

The sections were loaded onto a Leica autostainer (ST5020) for H&E staining. The sections went through xylene (×3) and a set of graded alcohols before washing in tap water. After washing, sections were placed in Haematoxylin z (CellPath, RBA-4201-00A) for 13 min before washing in tap water for 1 min. Next, the sections were placed in 1% acid alcohol and washed, and the nuclei were stained blue with Scotts tap water substitute (in house). The sections were washed with tap water for 1 min and then stained with Putts Eosin.

The following antibodies were used on a Leica Bond Rx autostainer: anti-ColV (Abcam, ab7046, RRID:AB_305723), anti-phospho-histone H2A.X (PH2AX; Cell Signaling Technology, 9718, RRID:AB_2118009, clone ID 20E3) and anti-p21 (Abcam, ab107099, RRID:AB_10891759). All FFPE sections underwent on-board dewaxing (Leica, AR9222) and epitope retrieval using appropriate epitope retrieval (ER) solution. Sections for ColV staining underwent retrieval using ER1 (Leica, AR9661), where ER1 solution was applied for 40 min at 95°C. Sections for PH2AX and p21 underwent retrieval using ER2 solution (Leica, AR9640) for 20 min at 95°C. For ColV and PH2AX, sections were then rinsed with wash buffer (Leica, AR9590) before peroxidase block was performed using an Intense R kit (Leica, DS9263) for 5 min. Sections were rinsed with wash buffer before application of primary antibodies at an optimised dilution (ColV, 1:200; PH2AX, 1:120) for 30 min. Sections were rinsed with wash buffer, and all had rabbit envision secondary antibody (Agilent, K4003) applied for 30 min. For p21, sections were rinsed with wash buffer (Leica, AR9590) before peroxidase block was performed using an Intense R kit (Leica, DS9263) for 5 min. Sections were rinsed with wash buffer before applying blocking solution applied from the Rat ImmPRESS kit (Vector Labs, MP7444-15) for 20 min. Sections were rinsed with wash buffer (Leica, AR9590) before anti-p21 antibody application at 1:250 dilution. The sections were rinsed with wash buffer before application of rat ImmPRESS secondary antibody for 30 min. For all stains, sections were rinsed with wash buffer, visualised using 3,3'-diaminobenzidine (DAB) and counterstained with Haematoxylin in the Intense R kit.

Sections for BrdU staining were initially dewaxed and underwent epitope retrieval using EDTA-based high PH retrieval solution (Agilent, K8004). Sections were placed into a pre-treatment (PT) module with the retrieval solution heated to 97°C for 20 min and then washed in flex wash buffer (Agilent, K8007) for 10 min prior to being loaded onto the Agilent Autostainer link48. A peroxidase blocking solution (Agilent, S2023) was applied to each section for 5 min before rinsing with wash buffer. Next, mouse Ig block (Vector Labs, MKB-2213) was placed onto each section for 20 min before rinsing with wash buffer. Anti-BrdU antibody solution (BD Biosciences, 347580, RRID:AB_10015219, clone ID B44) was applied to each section at 1:250 dilution for 30 min at room temperature. The sections were rinsed with wash buffer before application of mouse envision (Agilent, K4001) for 30 min. The sections were rinsed with wash buffer, liquid DAB (Agilent, K3468) was applied for 10 min, and then the reaction was terminated with water. The sections were removed from the autostainer, washed in tap water and then counterstained with Haematoxylin z (CellPath, RBA-4201-00A).

Staining for PSR was performed manually on FFPE sections that were dewaxed in xylene and taken through graded alcohols before washing in water. Rehydrated slides were stained for 2 h in PSR staining solution. The PSR staining solution was equal volumes of 0.1% Direct Red 80 (Sigma-Aldrich) and 0.1% Fast Green (Raymond A Lamb) (both in distilled water) combined in a 1:9 dilution with aqueous picric acid solution (VWR).

To complete the IHC, H&E and PSR staining, sections were rinsed in tap water, dehydrated through graded ethanol solutions and placed in xylene. The stained sections were coverslipped in xylene using DPX mountant (CellPath, SEA-1300-00A).

### Slide scanner and HALO

Stained slide sections were scanned at 20× magnification (resulting in a resolution of 0.503 µm/pixel) using a Leica Aperio AT2 slide scanner. Scanned images were analysed blindly using HALO Image analysis software (V3.1.1076.363; Indica Labs). Liver and tumour boundaries were manually defined using the HALO software. Classifiers were applied to identify tissues (organs, healthy regions of liver and tumours) and were analysed for the percentage of positive cells (γH2AX) or positive tissue area (γH2AX, BrdU, p21, ColV and PSR).

### Statistics and reproducibility

Sham-irradiated (CT-only controls) were used, and animal technicians were unaware of therapy group to prevent bias during husbandry and tissue sampling. Automated analysis and anonymised mouse identification numbers were used to prevent bias during the outcome analysis. GraphPad Prism software (version 10.3.1) was used for all statistical analyses. The Shapiro–Wilk normality test was used to assess for normal distribution of data. Data with a normal distribution were analysed using a two-tailed unpaired *t*-test. For data with more than two groups, a one-way ANOVA with multiple comparisons was used. For data where two groups are split between two independent variables, two-way ANOVA with multiple comparisons was used. For data that did not follow a normal distribution because the *N* was too small, ranked tests (e.g. a two-tailed Mann–Whitney test and Kruskal–Wallis test) were used. All data are presented as mean±s.e.m; *N* refers to the number of biological replicates, and *P*≤0.05 was considered significant. Figures were constructed using Scribus v1.4.7 (GNU general public license), Photoshop v12.1, Aperio ImageScope v12.4.0.5043 and GraphPad Prism 10 software. For representative images, processing adjustments were performed equally.

As this is the first report of the development of this model system, no statistical methods were used to pre-determine sample sizes for therapeutic studies, but our sample sizes in the orthotopic models are similar to those reported in previous publications (Leslie et al., 2022; Torrens et al., 2021; Kabarriti et al., 2019). Biological replicate sizes were chosen taking into account the observed variability in pilot and prior studies in treatment cohorts. For all experiments, animals/sample assignment was matched for age and within mouse batch from an external supplier and were assigned to experimental arm based upon randomly assigned mouse identification markings. Batched staining and analyses alongside controls were used throughout. The investigators were aware of groups for the *in vivo* experiments. All subsequent tissue handling and analysis was performed unaware of experimental group (anonymised identifiers) and/or performed using standardised automated analyses where possible. Quantitative image analysis was performed unaware of treatment. Aside from a single round of clonogenic *in vitro* assays, which were excluded owing to colony clumping preventing quantification, no data were excluded, except where explicitly stated in the figure legends; specifically, all mice entering the orthotopic and irradiation studies had a demonstrable tumour on CT prior to irradiation/sham, and none were excluded from analysis in the follow-up study.
